# Complete Genome Sequence of Escherichia coli Myophage Minorna

**DOI:** 10.1128/MRA.00533-19

**Published:** 2019-06-06

**Authors:** Katherine Rogers, Lorna Min, Heather Newkirk, Mei Liu, Jolene Ramsey

**Affiliations:** a Center for Phage Technology, Texas A&M University, College Station, Texas, USA; Queens College

## Abstract

The Gram-negative bacterium Escherichia coli causes many diseases, and antibiotic resistance has become a problem for their treatment. Bacteriophages may present a viable treatment alternative. Here, the complete genome sequence of E. coli-infecting myophage Minorna is presented. Proteins needed for replication, morphogenesis, and lysis were identified in the Minorna coding sequence.

## Abstract (precis)

The Gram-negative bacterium Escherichia coli causes many diseases, and antibiotic resistance has become a problem for their treatment. Bacteriophages may present a viable treatment alternative.

## ANNOUNCEMENT

The Gram-negative bacterium Escherichia coli causes serious human diseases, including bloodstream and urinary tract infections (1). The rate of antibiotic resistance in E. coli strains is rapidly rising (2), and bacteriophage therapeutics are the new frontier in combating this threat (3). Here, we describe the complete genome of a new E. coli myophage, Minorna.

Bacteriophage Minorna, against E. coli JE-1 carrying the plasmid pRA1 (RA1::Tn*5* Sq^r^), was isolated from the creek near Northgate Park in College Station, TX (4). The water sample was processed through a 0.22-μm filter. Luria broth or agar (BD) was used for growing the host and phage under aerobic conditions at 37°C, as described by Adams (5). To determine phage morphology, samples were negatively stained with 2% (wt/vol) uranyl acetate and viewed by transmission electron microscopy at the Texas A&M Microscopy and Imaging Center. Minorna genomic DNA was prepared as previously described (6) and sequenced on an Illumina MiSeq instrument with 250-bp paired-end reads using v2 500-cycle chemistry from an Illumina TruSeq Nano LT kit library. The 339,988 sequence reads from the index containing the phage genome were quality controlled using FastQC (http://www.bioinformatics.babraham.ac.uk/projects/fastqc/) and assembled using SPAdes v3.5.0 (7) with 687-fold contig coverage after trimming using the FastX toolkit 0.0.14 (http://hannonlab.cshl.edu). PCR (with primers 5′-CGCGCAGCGTAGCATATAAT-3′ and 5′-GAGTTACCTGAACGTAGCGAC-3′) and Sanger sequencing confirmed that the contig was complete. Genes were called using GLIMMER v3.0 and MetaGeneAnnotator v1.0 (8, 9), and tRNA genes were screened with ARAGORN v2.36 (10). The gene functions were predicted with InterProScan v5.22 (11), BLAST v2.2.31 against the NCBI nr (12) and UniProt Swiss-Prot and TrEMBL databases (13), LipoP (14), and TMHMM v2.0 (15). Rho-independent termination sites were annotated using TransTermHP (http://transterm.cbcb.umd.edu/). All analyses were run using default parameters in the Galaxy (16) and Web Apollo (17) instances hosted by the Center for Phage Technology (https://cpt.tamu.edu/galaxy).

Bacteriophage Minorna is a myophage with a 43,264-bp genome. The G+C content is 51%, matching the G+C content of its host. There are 57 protein-coding genes, 26 with predicted functions, and no tRNAs, with an overall 94% coding density. Minorna was compared with other phages by progressiveMauve2.4.0 (18) and shares similarity with many Klebsiella phages, including its highest nucleotide sequence identity, at 80%, with KPV811 (GenBank accession number KY000081) and its highest amino acid similarity at 84% identity to *Klebsiella* phages KpV48 (KX237514), both T1-like phages. Consistent with the packaging mechanism of phage T1, headful packaging for Minorna was predicted by PhageTerm (19).

A complete lysis cassette is present at the beginning of the genome, including an endolysin with two predicted transmembrane domains (GenBank accession number QBP07053), a holin with one transmembrane domain (QBP07054), and a unimolecular spanin (QBP07055) similar to the T1 phage unimolecular spanin, which is associated with both bacterial membranes (20, 21). The morphogenesis proteins identified are also grouped together and span from the large (QBP07058) and small terminase subunits through tail proteins, internal core protein, major capsid protein, and portal protein (QBP07071). The internal core protein (QBP07063) is the largest protein, at 1,232 residues. Encoded proteins for replication and transcription include DNA helicase (QBP07093), DNA primase (QBP07096), DNA polymerase (QBP07090), and RNA polymerase (QBP07075). There are no identified introns or frameshifts.

### Data availability.

The genome sequence and associated data for phage Minorna were deposited under GenBank accession number MK598851, BioProject accession number PRJNA222858, SRA accession number SRR8869232, and BioSample accession number SAMN11360441.
